# Epigenetic N6-methyladenosine modification of RNA and DNA regulates cancer

**DOI:** 10.20892/j.issn.2095-3941.2019.0347

**Published:** 2020-02-15

**Authors:** Zhixian Liang, Reilly L. Kidwell, Haijing Deng, Qi Xie

**Affiliations:** ^1^School of Life Sciences, Westlake University, Hangzhou 310024, China; ^2^Institute of Biology, Westlake Institute for Advanced Study, Hangzhou 310024, China; ^3^Division of Regenerative Medicine, Department of Medicine, University of California San Diego, San Diego, CA 92037, USA; ^4^University of California San Diego, School of Medicine, La Jolla, CA 92037, USA

**Keywords:** N6-methyladenosine, RNA methylation, DNA methylation, cancer, therapeutic targets

## Abstract

The biological roles of N6 methylation of nucleic acids have been extensively studied. Adenine methylation of RNA is the most prevalent RNA modification and has widespread effects on RNA splicing, translation, localization, and stability. Aberrant dynamic regulation of RNA N6-methyladenosine (m6A) has been reported in numerous human diseases, including several cancers. In recent years, eukaryotic DNA N6-methyladenosine (6mA) has also been reported and implicated in cancer progression and tumorigenesis. In this review, we summarize the contributions of N6-methyladenosine modification to cancer biology and pathogenesis in the context of both RNA and DNA. We also highlight the clinical relevance of targeting these modifications as a therapeutic strategy for cancer.

## Introduction

Epigenetic modifications have been implicated in diverse biological activities including mammalian development, longevity, and aging^1–3^. These modifications are chemical alterations in nucleic acids and associated proteins that occur without changing the DNA sequence^4^, and mainly include 1) DNA methylation and 2) histone modifications, such as lysine and arginine methylation, phosphorylation, acetylation, ubiquitylation, and sumoylation. Recently, RNA modifications have been identified as essential post-transcriptional regulators of gene expression^5^, and RNA N6-methyladenine (m6A) has been found to be the most abundant mRNA modification in mammalian cells^6^. The functional effects of m6A on RNA are regulated by dynamic interactions among associated methyltransferases (“writers”), demethylases (“erasers”), and binding proteins (“readers”). Localized aberrant base methylations frequently appear in disease-carrying genomes^7^, and aberrant regulation of m6A has been implicated in tumor initiation and progression through increased expression of oncogenes and/or silencing of tumor suppressor genes^8^.

Previous investigations into DNA methylation have focused on 5-methylcytosine^9,10^; however, increasing evidence has recently substantiated a role of DNA N6-methyladenosine (6mA) in transcriptional regulation. The 6mA alteration is the most abundant DNA modification, and it plays essential roles in DNA replication, repair, transposition, and transcription in prokaryotes^11–14^. Functional studies of 6mA in eukaryotes have long remained limited^15–18^; however, the development and utilization of highly sensitive detection techniques, including 1) 6mA dot blotting^19^, 2) ultra-performance liquid chromatography-tandem mass spectrometry^20^, and 3) genome analysis tools such as methylated DNA immunoprecipitation-sequencing (MeDIP-Seq)^21,22^ and single-molecule real-time sequencing (SMRT-Seq)^23,24^ have facilitated the discovery of 6mA in several eukaryotes, including *Chlamydomonas*^22^, *C. elegans*^23^, *Drosophila melanogaster*^25^, zebrafish^26^, mice^27^, rats^28^, pigs^26^, and humans^24,28,29^. Recently, our laboratory and others have demonstrated that 6mA is involved in various biological and disease processes in mammalian genomes, including embryonic stem cell differentiation, neurologic responses to environmental stress, and tumorigenesis^27,29–31^.

In this review, we provide a broad overview of the biological features of N6-methyladenosine modification in DNA and RNA, and summarize the roles of these alterations in cancer development and progression. We further highlight the exciting translational potential and clinical relevance of targeting N6-methyladenosine modifications and/or associated regulatory “writer”, “eraser”, and “reader” proteins to yield novel clinical therapies.

## The roles of RNA m6A in human cancer

**Figure 1:**
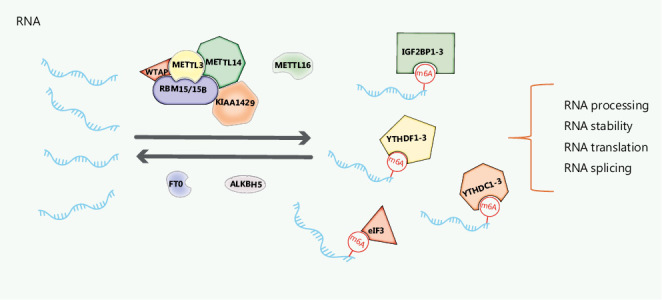
Regulators of RNA m6A. RNA methylation of m6A is mediated by the METTL3–METTL14 complex, including METTL3, METTL14, WTAP, RBM15/15B, and KIAA1429, or METTL16 (“writers”). FTO and ALKBH5 function as m6A demethylases (“erasers”). YTHDC1–2, YTHDF1–3, eIF3, and IGFBP1–3 (“readers”) recognize and bind m6A, thereby regulating RNA processing, stability, translation, and splicing in an m6A-dependent manner.

In mammalian cells, m6A is recognized as the most pervasive, abundant, and conserved internal modification in mRNAs, non-coding RNAs, and ribosomal RNA^32^. m6A has been detected adjacent to stop codons, long internal exons, transcription start sites, and the 5′ untranslated region (UTR) in mRNA^33^. m6A modification is tissue specific and dynamically regulated at different developmental stages by a series of enzymes and proteins: 1) methyltransferases (“writers”), 2) demethylases (“erasers”), and 3) m6A binding proteins (“readers”) (**Figure 1**)^33–35^. Abnormal expression and dysfunction of these modifiers has been observed in numerous cancers and has been implicated in the development and progression of these malignancies^8,36–42^.

### Methyltransferases (“writers”)

Proteins in the methyltransferase like 3 (METTL3)-containing methyltransferase complex serve as “writers” that catalyze the formation of m6A on specific target RNAs within nuclear speckles. Within this complex, METTL3 is the catalytically active subunit, alongside its cofactor, methyltransferase-like 14 (METTL14), which plays an essential structural role in substrate recognition^43,44^. The METTL3–METTL14 complex methylates RNA through binding the specific consensus motif DRACH (where D is A, G, or U, and H is A, C, or U)^45^.

Additional proteins also contribute to the function and activity of the methyltransferase complex by altering methyltransferase activity, targeting the complex to nuclear speckles, and mediating target recognition. Wilms’ tumor 1 associating-protein (WTAP) regulates m6A activity and is required for accumulation of METTL3 and METTL14 in nuclear speckles. WTAP also increases the binding capacity of METTL3, thus regulating recruitment of the complex to mRNA targets^46^. RNA binding protein motif 15 (RBM15) and its paralogue, RBM15B, also facilitate m6A recruitment to specific RNA sites, particularly within the RNA X-inactive specific transcript (XIST), thus mediating m6A formation and transcriptional silencing^47^. Recently, KIAA1429, another member of the methyltransferase complex, has been shown to regulate m6A modification of ID2 mRNA and has been implicated in cellular migration and invasion in hepatocellular carcinoma^48^. Another writer METTL16 has recently been identified as a methyltransferase of U6 spliceosomal small nuclear RNA and a regulator of S-adenosylmethionine homeostasis^49,50^. Interestingly, METTL16 targets a distinct subset of m6A sites from the METTL3 complex; these sites are primarily localized to introns or intron-exon boundaries^49^.

The METTL3–METTL14 complex mediates expression of tumor-related genes *via* m6A modification of associated mRNA, thus controlling cancer stem cell pluripotency, tumor initiation, epithelial-mesenchymal transformation (EMT), angiogenesis, and the DNA-damage response. m6A within the coding sequence of the EMT regulator Snail triggers polysome-mediated translation of Snail mRNA in cancer cells, and deletion of METTL3 impairs cancer cell migration, invasion, and EMT^51^. METTL14 regulates the m6A levels of key transcripts relating to EMT and angiogenesis, thus resulting in increased gene expression and subsequent tumor-associated angiogenesis and cancer progression^52^. METTL3 also participates in DNA repair *via* rapid and transient induction of m6A in response to DNA damage. This process is accomplished by the specific catalytic activity of METTL3, which helps DNA polymerase κ localize to sites of ultraviolet-light-induced DNA damage^53^.

Upregulation of one or more components of the methyltransferase complex has been observed in several cancers, and is associated with poor clinical outcomes. For example, high expression of METTL3 and METTL14 has been observed in acute myelocytic leukemia (AML) and found to mediate transformation of malignant myeloid hematopoietic cells^37,38^. Deletion of METTL3 or METTL14 delays leukemia progression, thus suggesting that m6A methyltransferases may be attractive candidates for therapeutic targets in AML^54^. Overexpression of METTL3 or METTL14 also promotes tumor progression in solid cancers. METTL14 suppresses P2RX6 activation, thus promotes cell migration and invasion in renal cancer^55^. METTL3 acts an oncogene that maintains SOX2 expression through an m6A–IGF2BP2-dependent mechanism in colorectal carcinoma^56^, and facilitates tumorigenicity and lung metastasis in hepatocellular carcinoma^57^. Finally, METTL3 overexpression promotes bladder cancer cell growth through activation of the AFF4/NF-κB/MYC signaling network^39^, and inhibition of METTL3 decreases malignant cell proliferation, invasion, and survival^58^. Concordantly, METTL3 overexpression is correlated with poor clinical prognosis in all these cancers. Together, these data suggest that METTL3 is a key driver of malignant transformation and tumorigenesis.

RNA methylation in non-coding RNAs, including microRNAs, long non-coding RNAs (lncRNAs) and circular RNAs, has also been linked to cancer cell proliferation and migration^59–63^. In colorectal carcinoma, m6A methylation of circNSUN2 mediates cytoplasmic export and enhances stability of HMGA2 mRNA, thus promoting cellular invasion and liver metastasis. Furthermore, METTL3 silencing increases nuclear circular RNA and decreases cytoplasmic export, thus demonstrating that intact METTL3–m6A binding capacity is necessary for the export function^60^. METTL3-regulated m6A methylation also increases nuclear accumulation of RP11, thus mediating downstream changes in the expression of Siah1–Fbxo45/Zeb1 and the development of colorectal cancer^61^. In nasopharyngeal carcinoma, METTL3-regulated m6A methylation is highly enriched within the lncRNA FAM225A and is also a key enhancer of RNA stability, promoting tumorigenesis and metastasis^62^. Furthermore, METTL3 accelerates pri-miR221/222 maturation in an m6A-dependent manner, thus promoting tumor proliferation in bladder cancer^59^. METTL3 may also be a target of non-coding RNA. Targeting of METTL3 by the non-coding RNA miR-4429 has been reported to prevent progression of gastric cancer by inhibiting m6A-dependent stabilization of SEC62^63^.

Of note, the role of METTL3–METTL14 in some cancers remains controversial. Methyltransferase expression has been associated with tumor suppression in several cancer types. Low m6A levels secondary to METTL14 mutation or decreased METTL3 expression are observed in 70% of endometrial cancers, and low m6A is associated with increased activation of oncogenic AKT signaling through translation inhibition of the AKT negative regulator PHLPP2, and mRNA stabilization of the AKT positive regulator mTORC2^64^. Similarly, low METTL3 expression activates mTOR pathways in clear cell renal cell carcinoma and is correlated with poor clinical outcomes^65^. In glioma, METTL3 inhibits growth, self-renewal, and tumorigenesis of glioma stem cells (GSCs) by regulating the expression of crucial genes (e.g., *ADAM19*)^66^. In contrast, increased expression of METTL3 and METTL14 has also been implicated in glioma resistance and progression. Visvanathan et al.^67^ have reported that METTL3 increases radiotherapy resistance by stabilizing SOX2 mRNA, and other work has demonstrated that METTL3- and METTL14-mediated m6A modification is crucial for maintenance of GSCs^66^. The disparity in these results may be partly due to the utilization of differing research models and the challenges in accurately modeling such heterogeneous tumors; however, further elucidation and confirmation of the true functional role of this complex in glioma remains a target of future exploration. Moreover, varying functions of m6A methyltransferases in different tumor types are likely to be attributable to differential genomic backgrounds and tumor-specific preferences for substrate selection.

### Demethylases (“erasers”)

In mammalian cells, m6A is also regulated by 2 demethylases: FTO and ALKBH5^68,69^. Removal of methyl groups from m6A by these “erasers” allows m6A RNA modification to be dynamic and reversible. FTO is highly expressed in several human cancers, and it enhances tumorigenesis and cell transformation^70^. Correspondingly, FTO deletion increases m6A methylation in oncogenes, thus resulting in recruitment of the “reader” binding protein YTHDF2, increased RNA decay, and sensitization of tumor cells to anti-PD-1 treatment. Upregulation of FTO is consistently observed in solid tumors. In melanoma, FTO promotes cancer cell growth and suppresses the effects of anti-PD-1 blockade immunotherapy, and inhibition of FTO in glioma suppresses cancer stem cell growth and self-renewal^66,71^. Furthermore, FTO promotes tumor cell proliferation, colony formation, and metastasis in breast cancer by mediating m6A demethylation in the 3′UTR of BNIP3 mRNA^72^. FTO has also been implicated in hematologic malignant transformation. FTO decreases m6A levels on the AML-associated genes *ASB2* and *RARA*, thus resulting in inhibition of all-trans-retinoic acid-induced AML cell differentiation and enhanced leukemogenesis^40^. Su et al.^73^ have also reported that FTO stabilizes oncogenic MYC/CEBPA mRNA *via* demethylation of m6A, thereby leading to rapid tumor growth. The authors have further identified a small molecule inhibitor of FTO, R-2HG, which decreases the proliferation and survival of tumor cells, thus suggesting that targeting m6A demethylases may be an effective therapeutic strategy for treating AML and possibly other cancers.

ALKBH5, the second m6A demethylase, is also associated with several cancers. ALKBH5 is highly expressed in GSCs and maintains tumorigenesis by sustaining expression of the transcription factor FOXM1^74^. ALKBH5-mediated m6A-demethylation of NANOG mRNA under hypoxic conditions also induces breast cancer stem cell phenotypes. Moreover, ALKBH5 promotes gastric cancer invasion and metastasis by decreasing methylation of the lncRNA NEAT1 and inhibits autophagy in epithelial ovarian cancers through upregulation of miR-7 and BCL-2^75,76^. Although both FTO and ALKBH5 belong to the AlkB family, they have differing substrate specificity for human cancers. It has been reported that this difference is attributable to differing active-site residues between these two enzymes, and that the substrate specificity of these enzymes can be switched by exchanging their active site sequences ^77,78^.

### m6A binding proteins (“readers”)

YTHDC1, YTHDC2, and YTHDF family proteins (YTHDF1, YTHDF2, YTHDF3), eukaryotic initiation factor 3 (eIF3), and IGF2BP family proteins (IGF2BP1, IGF2BP2, and IGF2BP3) have been reported to be m6A “readers” that specifically recognize and bind m6A in RNA and regulate downstream functions.

The highly conserved YTH family of proteins recognize target RNA *via* a YTH RNA-binding domain^79^ and can be divided into 3 major classes: DC1, DC2, and the DF family. YTHDC1 localizes to the nucleus and regulates m6A-related mRNA splicing^80^. Subsequently, mature m6A modified RNA undergoes cytoplasmic regulation by YTHDC2 and DF family proteins. Here YTHDC2 regulates translation efficiency and decreases the mRNA abundance of its targets^81^. YTHDF family proteins also interact with both YTHDC2 and one another, thereby enhancing the translational efficiency of RNA targets and regulating mRNA stability. Independently, YTHDF1 engages translation initiation factors, thus increasing mRNA translation efficiency, but also cooperatively interacts with 40S/60S ribosomal subunit proteins alongside YTHDF3 and consequently regulates mRNA translation^82^. YTHDF2 recruits the CCR4–NOT deadenylase complex, which facilitates RNA degradation, and also works in concert with YTHDF3 in mediating mRNA decay^83^. YTHDF3 thereby regulates mRNA binding specificity for both YTHDF1 and YTHDF2, and moderates the fate of m6A mRNA transcripts.

The multiprotein complex eIF3 also acts as an m6A “reader” and facilitates mRNA translation. Binding of eIF3 to a single m6A in the 5´UTR (acting as an m6A-induced ribosome engagement site) has been demonstrated to be necessary and sufficient for recruitment of the 43S complex and initiation of cap-independent translation^84^. Finally, the IGF2BP family proteins are a distinct family of cytoplasmic m6A readers that recognize and bind the GG(m6A)C sequence *via* their K homology domains. IGF2BP proteins have been shown to promote mRNA target stability and storage, and therefore have been implicated as key regulators of oncogene (e.g., *MYC*) expression^85^. More specifically, IGF2BP1 binds the 3′UTR of serum response factor (SRF) mRNA in an m6A-dependent manner and promotes SRF mRNA expression by impairing microRNA-dependent decay in several cancer cell lines. Conversely, few studies have attempted to elucidate the precise functions of IGF2BP2 and IGF2BP3 in cancer^86^.

The IGF2BP family proteins are not the only m6A “readers” implicated in tumor progression. Recent studies have demonstrated that YTHDF1 mediates m6A-induced translation of Snail mRNA, thereby promoting EMT^51^. Furthermore, YTHDF2 is overexpressed in AML and is required for tumorigenesis. YTHDF2 deficiency has been shown to increase AML cell sensitivity to tumor necrosis factor and to prime cells for apoptosis^42^. YTHDF2-dependent posttranscriptional silencing of SOCS2 also promotes progression in liver cancer^57^.

Intriguingly, binding proteins have also been shown to regulate tumor immunity. In dendritic cells, YTHDF1 binding increases translation of lysosomal cathepsins, which inhibit the cross-presentation of wild-type dendritic cells. YTHDF1 deletion therefore enhances antitumor immunity^87,88^. Further investigation of these proteins and their dynamic roles in cancer biology is necessary to deepen understanding of RNA methylation, and may provide additional insights into mechanisms of cancer pathogenesis and therapeutic strategy.

## The roles of DNA 6mA in human cancer

**Figure 2:**
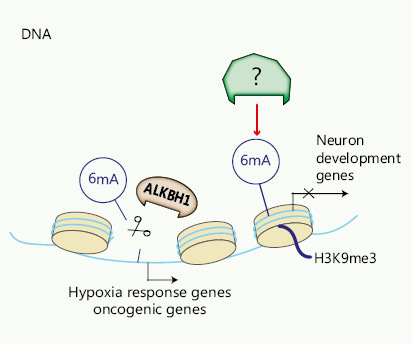
Regulators of DNA 6mA. DNA 6mA is demethylated by ALKBH1. ALKBH1 regulates expression of hypoxia response genes and oncogenic genes in a 6mA-dependent manner. The 6mA methylase requires further investigation.

Our laboratory and others have reported novel data implicating DNA 6mA modification in the development and progression of human cancers. Xiao et al.^29^ have observed decreased abundance of 6mA in primary gastric and liver cancer tissues, and this 6mA downregulation correlates with increased tumorigenesis. Recently, we have observed higher levels of 6mA in GSCs and primary glioblastoma than in normal human astrocytes, and have found that these higher levels are associated with disease progression^30^. DNA immunoprecipitation sequencing (DIP-seq), has illustrated that 6mA is enriched in intergenic regions, in agreement with findings from reports from other studies of mammalian cells^31,89,90^, and 6mA co-localizes with heterochromatic histone modification markers, predominantly H3K9me3 and H3K27me3. Furthermore, we identified ALKBH1 as a demethylase and dynamic regulator of 6mA in glioblastoma. Targeting ALKBH1 inhibits tumor growth and reduces stemness and tumorigenesis by down-regulating expression of hypoxia-associated genes and other tumor-associated genes. Interestingly, levels of both 6mA and the 6mA demethylase ALKBH1 are elevated in glioblastoma, thus suggesting that additional undiscovered DNA methyltransferases may also contribute to the high levels of 6mA in glioblastoma (**Figure 2**)^30^.

Xiao et al.^29^ have reported that N6AMT1 is a key 6mA methyltransferase observed in liver and gastric cancers; however, our investigations did not detect 6mA methytransferase activity either *in vivo* or *in vitro*. Moreover, biochemical and structural evidence demonstrates that N6AMT1 forms a complex with Trm112 and functions as a protein methyltransferase rather than a DNA methyltransferase^91,92^. Recently, Kweon et al.^90^ have reported that METTL4 catalyzes 6mA deposition and that inactivation of METTL4 results in diminished 6mA levels in mouse cells. Deposition of 6mA by METTL4 also triggers proteolytic destruction of sensor proteins (e.g., ASXL1) that have been linked to multiple cancers, including leukemia and glioblastoma. Additional investigations will probably be needed to confirm the identity of 6mA methyltransferases in human cells; however, extensive observations of 6mA upregulation in various malignancies suggest that 6mA may be a promising target for cancer therapeutics.

## Clinical applications of N6-methyladenosine

Because RNA methylation plays an extensive regulatory role in several cancers, profiling of RNA methylation has the potential to be used as a clinical tool. The prognostic value of RNA methylation has been reported in several studies. Survival analysis has demonstrated that clinical outcomes for patients with colorectal cancer are tightly correlated with expression levels of RNA methylation regulators^93^. Zhou et al.^65^ have demonstrated that deletion of m6A “writer” genes and copy number gain of “eraser” genes are independent risk factors for overall survival in renal clear cell carcinoma. Additional m6A regulators are also associated with decreased overall survival, including upregulation of the “reader” YTHDF1 in liver cancer^57^, and elevated IGF2BP1 expression in ovarian, liver, and lung cancers^86^. High expression of the “writer” METTL3 is associated with poor prognosis of patients with several cancers, including HCC, AML, and glioma^39,57,59,67,94^. One notable exception to this observed pattern across malignancies is low expression of METTL3 is associated with poorer clinical outcomes in renal clear cell carcinoma^65^.

For gliomas, m6A regulators are currently measured as biomarkers for patient classification into 1 of 2 subgroups, each of which confers a differential prognosis, World Health Organization grade, and isocitrate dehydrogenase mutation status. m6A regulator expression level also correlates with mesenchymal subtype and sensitivity to temozolomide^95^. Additionally, our previous work has identified the DNA demethylase ALKBH1 as critical to GSC survival, thus suggesting ALKBH1 as a promising target for cancer therapy. The “eraser” FTO is also a particularly promising therapeutic target for glioma and other cancers. Meclofenamic acid, a highly selective inhibitor of FTO, reduces GSC growth, self-renewal, and stemness in glioma^66^. Elevated levels of FTO are also associated with lower survival rates in patients with breast cancer^72^.

The many observed correlations between epigenetic methylation and disease prognosis suggest valuable translational potential. However, despite notable advances in the past several years, the challenges of tumor heterogeneity and increasing clinical demand will require further mechanistic investigations and development of successful inhibitors to bring these discoveries from bench to bedside.

## Conclusions and perspectives

Over the past several years, much progress has been made in understanding the regulators of DNA and RNA N6-methyladenosine and the mechanisms underpinning their contributions to cancer biology. These investigations have proven to have critical clinical relevance, because N6-methyladenosine modifications play a key role in cancer development and progression, and many associated regulators correlate with patient prognosis and clinical outcomes. Research in this field therefore holds much promise in identifying novel targets for cancer therapy (**Table 1**).

However, several outstanding challenges have hindered the ability to translate these targets into clinical practice to date. First, investigation of the role of DNA 6mA in tumor biology remains in its infancy, and the DNA 6mA methyltransferase remains to be identified in cancers. Although N6AMT1 has been reported to be a likely candidate, its recently published protein structure suggests that this enzyme is likely to be a protein lysine methyltransferase rather than a DNA 6mA methyltransferase^29^. METTL4 is another proposed candidate; however, further interrogation with additional techniques (e.g., *in vitro* methylation assays) is required to validate methyltransferase activity. Additionally, the role and identification of specific “readers” of 6mA remain unknown. Future investigations should focus on identification of these key players as well as on the exploration of the unknown interplay between 6mA and other epigenetic modifications (e.g., 5-methylcytosine, H3K9me3, and H3K27me3). Furthermore, understanding of how these interactions contribute to the regulation of gene expression in the setting of tumorigenesis will be crucial to the development of novel clinical interventions.

Second, for RNA 6mA, the mechanisms underpinning how a single methylation regulator may have various functions within different tumors remain unknown. We have suggested that variability in mutations and the tumor microenvironment may contribute to regulator substrate preference and subsequent function; however, additional data are needed to confirm or expand upon this conjecture. Advanced disease profiling techniques, including tissue-specific conditional knockout mouse models and patient-derived xenograft organoids will probably be useful in addressing these questions.

Third, tools enabling precise editing of m6A have been lacking so far; therefore, m6A function has been studied only indirectly through manipulation of methyltransferase and demethylase levels. However, the vast biological implications of RNA 6mA have driven development of new technological advances. Recent exciting work has promised the development of new m6A editing tools using CRISPR–Cas9 fused with METTL3–METTL14 or ALKBH5/FTO^96^. With this technology, m6A “writers” and “erasers” can be designed and programmed with a guide RNA to both functionally compare and demethylate specific gene regions, thereby allowing precise editing of an m6A site without alteration of surrounding regions. Advances in genetic tools are expected to greatly improve characterization of the regulators of m6A and facilitate further understanding of the complex and dynamic contributions of m6A in cancer. Collectively, these challenges, although difficult, are not insurmountable, but will need to be addressed to allow translation to clinical settings.

## Figures and Tables

**Table 1:** Roles of “writers”, “erasers”, and “readers” of m6A and 6mA in different tumors

	Target	Class	Name	Tumor	Function	Reference
RNA	mRNA	“Writers”	METTL	Glioma	Maintain stem cell pluripotency	^67^
					Inhibit stem cell pluripotency	^66^
				AML	Maintain stem cell pluripotency	^37^
					Promote tumor progression	^54^
				CRC	Promote tumor progression	^56^
				Bladder cancer	Promote tumor progression	^39^
					Promote tumorigenesis	^58^
				HCC	Promote tumor progression	^57^
					Epithelial-mesenchymal transition	^51^
					DNA damage response	^53^
				Renal cancer	Suppress tumor progression	^65^
			METTL14	AML	Maintain stem cell pluripotency	^38^
				Glioma	Inhibit stem cell pluripotency	^66^
				Renal cancer	Promote tumor progression	^55^
					Epithelial-mesenchymal transition, angiogenesis	^52^
				Endometrial cancer	Suppress tumor tumorigenicity	^64^
		“Erasers”	FTO	AML	Maintain stem cell pluripotency	^40^
					Promote tumor progression	^73^
				Melanoma	Promote tumor progression	^71^
				Breast cancer	Promote tumor progression	^72^
				Glioma	Maintain stem cell pluripotency	^66^
			ALKBH5	Glioma	Promote tumorigenesis	^74^
		“Readers”	YTHDF2	AML	Maintain stem cell pluripotency	^42^
				Liver cancer	Promote tumor progression	^57^
			YTHDF1	Melanoma	Suppress antitumor immunity	^87^
			IGFBP1	Ovarian cancer; liver cancer; lung cancer	Promote tumor progression	^86^
	Non-coding RNA	“Writer”	METTL3	Bladder cancer;	Promote tumor progression	^59^
				Gastric cancer	Promote tumor progression	^63^
		“Eraser”	ALKBH5	Gastric cancer	Promote tumor progression	^75^
DNA	DNA	“Writer”	N6AMT1	Gastric cancer; liver cancer	Inhibit tumorigenesis and metastasis	^29^
		“Eraser”	ALKBH1	Glioblastoma	Maintain cell viability and stemness properties	^30^
				Gastric cancer; liver cancer	Promote tumorigenesis and metastasis	^29^

AML, acute myelocytic leukemia; CRC, colorectal carcinoma; HCC, hepatocellular carcinoma.
